# Differential gene expression in the endometrium on gestation day 12 provides insight into sow prolificacy

**DOI:** 10.1186/1471-2164-14-45

**Published:** 2013-01-22

**Authors:** Hao Zhang, Shouqi Wang, Manqing Liu, Ailing Zhang, Zhenfang Wu, Zhe Zhang, Jiaqi Li

**Affiliations:** 1 Guangdong Provincial Key Lab of Agroanimal Genomics and Molecular Breeding, College of Animal Science, South China Agricultural University, Guangzhou, Guangdong, 510642, China

## Abstract

**Background:**

Erhualian pigs, one of Chinese Taihu pig breeds, are known to have the largest recorded litter size in the world. A lower prenatal death rate is the major contributing factor to the prolificacy of Taihu pigs. Cross-breeding experiments have demonstrated that Taihu sows exhibit a strong maternal effect and that their large litter sizes are mainly caused by maternal genes. The growth and development of porcine embryos on gestation day (GD) 12 are dependent on histotroph secreted by endometrium. Embryonic loss of Taihu pigs on GD12 is lower than that of Western pigs. Here, endometrial samples were collected from pregnant Erhualian sows (parity 3) and Landrace × Large White (LL) sows (parity 3) on GD12. Digital gene expression profiling (DGE) was used to measure the gene expression in the endometrium of the two breeds.

**Results:**

A total of 13,612 genes were differentially expressed between the two breeds (*P* < 0.001, *FDR* < 0.001). Gene Ontology (GO) analysis showed that the differential genes involved in reproduction and growth. Pathway analysis revealed that the differentially expressed genes significantly enriched in 24 KEGG pathways. Quantitative real-time RT-PCR confirmed the differential expression of eight genes. Analyses of the differentially expressed genes suggested possible reasons for the difference in embryonic survival ratio between the two breeds. Specifically, these findings point to a higher ratio of PGE_2_:PGF_2α_ in the endometrium of Erhualian pigs, which facilitates the establishment and maintenance of pregnancy. We also suggest that the differences in the uterine environment lead to higher uterine capacity in Erhualian pigs.

**Conclusions:**

The DGE expression profiles of Erhualian and LL endometrium demonstrated differential expression of genes. Our results will increase understanding of the molecular mechanisms of the low rate of embryonic loss in Chinese Taihu pigs, facilitate the identification of major genes that affect litter size, and be valuable for porcine transcriptomic studies.

## Background

Chinese Taihu pigs are highly prolific; the Erhualian (ER), one of the Taihu pigs, is known for producing the highest recorded litter sizes in the world [1]. Litter size is influenced by many factors, such as the boar, season, and nutrition. However, it has been demonstrated that these factors do not account for the prolificacy of Meishan pigs, which are another breed of Chinese Taihu pig [2]. In addition, Meishan sows are little affected by the factors involved in stillbirth [3]. Taihu pigs express a high level of maternal heterosis in litter size when used in crosses with Western pig breeds [4,5]. Studies have indicated that the large litter sizes of Meishan pigs are due to genes acting in the dam [6,7]. The ER sows can give birth to more than 15 piglets per litter, even when the coefficient of inbreeding is as high as 0.25 [8]. These findings indicate that the desirable alleles related to litter size are preponderant in Taihu sows.

Embryonic loss is one of the major barriers to large litter size [2,9]. It is estimated that approximately 20-30% of embryonic death occurs during gestation days (GD) 11–12 [10]. The embryonic survival rate does not differ among pig breeds until GD11, but it is elevated on GD12 in Meishan pigs when compared with Landrace × Large Yorkshire (LL) pigs [7,11]. At this stage, the blastocysts undergo dramatic morphological changes, developing from an 11–50 mm tubular structure into a 100 mm filamentous structure. The rapid changes in shape and size caused by the elongation of porcine blastocysts are not a result of cellular hyperplasis but cellular rearrangements and remodeling of the trophectoderm [12]. These changes coincide with the synthesis and release of maternal-fetal recognition signals (estrogen) and cytokines required for the establishment of pregnancy [13-15]. Porcine conceptuses initiate the secretion of estrogen on GD10-15 [16], although Meishan embryos are smaller and contain fewer cells when they initiate steroidogenesis and begin to elongate [17]. Meishan conceptuses also secrete less estrogen into the uterine luminal fluid and elongate to a reduced length [18] and diameter [17,19] when compared with Large White conceptuses.

The level of estrogen in porcine uterine flush samples is determined primarily by the amount of estrogen secreted by the embryos [20]. The estrogen level in the uterine lumen will have multiple effects on the embryonic survival rate. Firstly, the estrogen level may affect placental weight and survival of the conceptus. When Meishan gilts were treated with estrogen on GD12 or GD13, placental weights were increased significantly (*P* < 0.05); litter size was not affected significantly (*P* > 0.05) but it tended to decrease [21]. However, others have shown that placental weights are negatively correlated with litter size (*P* < 0.05) [22] and uterine capacity at GD105 [23] (*P* < 0.01) in Western breeds. The non-significant result in the former study [21] may have been a consequence of smaller sample size. Secondly, embryonic estrogen, as an embryo-maternal recognition signal, can change uterine secretion of histotroph [24]. The lower amount in Meishan embryos may cause a more gradual change of the gravid uteri, which decreases the negative impact that faster-developing embryos could have on their slower-developing littermate embryos [25,26].

Endometrial synthesis of prostaglandins (PG) is essential for the establishment and maintenance of pregnancy in pigs [27,28]. During maternal recognition of pregnancy around GD12, PGF_2α_, which is synthesized mainly by the endometrium [15], has a luteolytic effect, while PGE_2_ can antagonize this effect [29,30]. The secretion of PGF_2α_ is redirected from the uterine venous drainage (endocrine) during luteolysis to the uterine lumen (exocrine) at the time of maternal recognition of pregnancy. Studies have shown that the PGE_2_:PGF_2α_ ratio is crucial for the regulation of the estrous cycle, and the establishment and maintenance of pregnancy [31,32]. The sum of PGE_2_ and PGF_2α_ and their ratio were higher in Meishan sows than that in Large White pigs [33].

On GD12, the placenta (trophectoderm) has not yet formed, the conceptus is free-floating and not attached to the endometrium [10,12], hence embryonic growth and development is dependent on histotroph in the uterine lumen. Histotroph includes hormones, growth factors, and transport proteins [34]. The uterine histotroph is synthesized and secreted primarily by the epithelia of the maternal uteri during early pregnancy [35]. Experiments have demonstrated that embryonic growth and development are affected by the environment of the uterine lumen [18,36]. In the present study, we detected the differentially expressed genes in the endometrium of ER and LL pigs on GD12 by digital gene expression profiling (DGE) using an Illumina Genome Analyzer platform. This work will be helpful for understanding the molecular basis of different prolificacy between Chinese Taihu and Western pigs.

## Results

### DGE libraries

**Figure 1:**
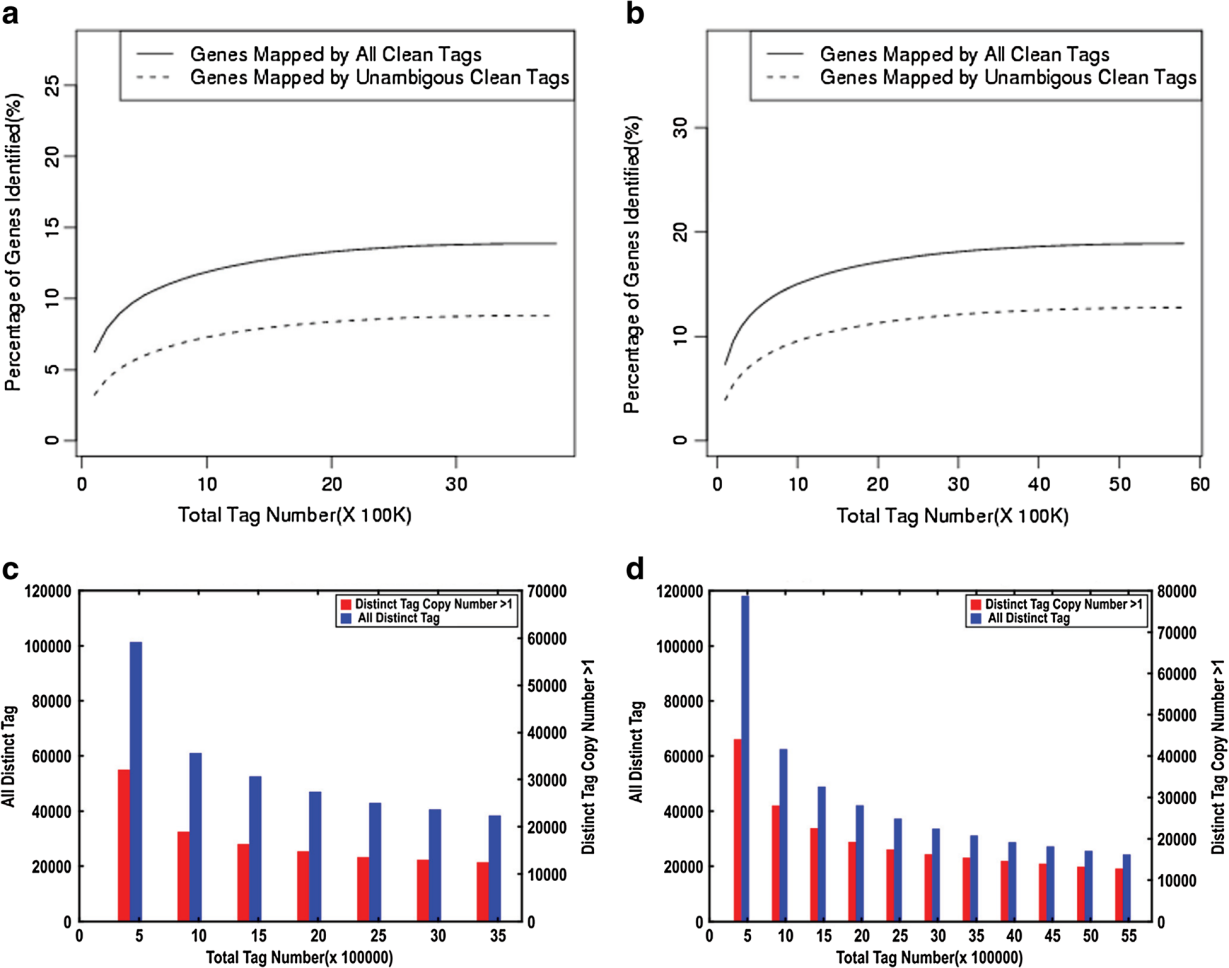
**Saturation analysis.** ER: (**a**) and (**c**); LL: (**b**) and (**d**). Saturation analysis of DGE libraries showed that new emerging genes and unique tags were gradually reduced with increasing total sequence tags when the library of total sequencing tags was large enough. Percentage of genes (**a** and **b**) or unique tags (**c** and **d**) identified gradually decreased with increasing total tags when the number of tag sequenced was high enough.

Pools of cDNA from the GD12 endometrium of three LL (parity 3) and three ER (parity 3) sows were used to construct the two DGE libraries. Global gene expression profiles were obtained by massive parallel sequencing using the Illumina DGE system. The raw data of the DGE libraries were filtered to obtain clean tags before further analysis. The major characteristics of the two DGE libraries are described in Table 1 and Additional file 1: Figure S1. A total of 9,514,757 tags, including 3,723,534 for ER and 5,791,223 for LL, were obtained by sequencing. The clean tags consisted of 3,448,173 in ER and 5,496,993 in LL, which contained 400,769 and 493,761 unique tags in ER and LL, respectively. In both raw tag libraries, more than 92% of the tags were detected more than once within each library. The distribution of tags revealed that high-expression tags (copy number >100) represented the majority of sequences detected, whereas the low-expression tags (copy number <5) had the greatest sequence diversity. Tags that represented less than 3% of the total categories of tags identified in this study accounted for more than 61% of the total number of tags. Conversely, tags that represented more than 60% of the total tag types accounted for less than 7% of the total tags. This indicates that only a small number of mRNAs are expressed abundantly and that the vast majority of mRNAs are present at low level. Saturation analyses of ER and LL (Figure 1) demonstrated that the number of newly identified unique tags and genes decreased as the total number of sequencing tags increased, which shows that the DGE libraries were becoming saturated, and validates the integrity of the library for use in further analysis.

**Table 1:** Major characteristics of DGE libraries and tag mapping to the integrated transcript database

	**ER**		**LL**	
	**Total Tags**	**Unique Tags**	**Total Tags**	**Unique Tags**
Raw Data	3,723,534	400,769	5,791,223	493,761
Low Quality Tag	6,749	5,681	68,587	48,070
Adaptors^1^	0	0	0	0
Tag CopyNum <2	268,612	268,612	225,643	225,643
Clean Tags	3,448,173	126,476	5,496,993	220,048
CopyNum > =2	3,448,173	126,476	5,496,993	220,048
CopyNum >5	3,225,291	45,507	5,124,374	87,931
CopyNum >10	3,102,559	29,281	4,884,200	56,377
CopyNum >20	2,944,609	18,456	4,552,034	33,675
CopyNum >50	2,659,626	9,541	3,950,181	14,721
CopyNum >100	2,352,245	5,212	3,399,787	6,871
3′ tag mapping				
Tags Mapped to Gene^2^	3,279,258	98,673	4,218,582	159,029
Unambiguous Tags Mapped to Gene^3^	2,289,131	77,128	3,002,699	130,494
Tags Mapped to Mitochondrion	355	39	562	55
Tags Mapped to Genome	90,041	16,150	170,475	38,077
Unknown Tags	78,519	11,614	1,107,374	22,887

^1^There is only adaptor but no tag sequence in the reads. ^2^Tag Mapped to Gene represents the number of tags mapped to the reference library. ^3^Unambiguous tags mapped to Gene represents the number of tags mapped to a gene in the reference library.

### Tag mapping

Three databases (GenBank + EMBL + TIGR) were used to generate an integrated reference library for DGE tag mapping and sequence annotations. The tags in the reference library consisted of CATG, the recognition site for *Nla*III, in conjunction with the next 17 nt sequences that were created by *Mme*I. One mismatch was allowed for DGE tag mapping to allow for potential polymorphisms between samples. This generated 649,443 reference tags, which corresponded to 425,980 unambiguous reference tags in the integrated reference library. Together, 95.10% and 76.74% of the clean tags and 78.02% and 72.27% of the unique clean tags were mapped to the reference library for ER and LL, respectively; 66.39% and 54.62% of the total clean tags and 60.98% and 59.30% of the unique clean tags were mapped unambiguously to the integrated reference library for ER and LL, respectively. In total, 12.80% and 17.33% of the unique tags were mapped to the mitochondrial genome and nuclear non-coding genome sequence, respectively. Other DGE unique tags (approximately 9.18% and 10.40% for ER and LL, respectively) were not mapped to the integrated reference library. These unknown tags probably arose from incomplete reference tag libraries. Tag position analyses (Additional file 2: Figure S2) indicated that the most DGE tags that matched the reference tags were close to the 3^′^ end of the transcripts. DGE based on Illumina sequencing was able to discriminate the tags from the sense and antisense strands of DNA. We found that 13,966 genes (2,210 NCBI, 1,153 GenBank, 3,575 TC, 5,951 Unigene and 1,075 ENSEMBL) had antisense transcripts for ER (Additional file 3: Table S1)), and 11,033 genes (1,542 NCBI, 1,437 GenBank, 2,980 TC, 4,129 Unigene and 945 ENSEMBL) for LL (Additional file 4: Table S2). In total, 16,150 and 38,077 unique tags were mapped to the non-coding nuclear genome for ER (Additional file 5: Table S3) and LL (Additional file 6: Table S4), respectively, which suggests that novel transcripts may exist close to these tags.

### Identification and analysis of differentially expressed genes

The tag number obtained via DGE reflects the level of expression of the transcripts represented by those tags. All the clean tags were mapped to the reference sequences; the number of unambiguous clean tags for each gene was calculated and normalized to tags per million (TPM). By comparing the normalized DGE profiles between ER and LL, we obtained the global transcriptional difference between ER and LL. The results showed that 13,612 genes were significantly differentially expressed between the breeds (Additional file 7: Table S5); 5,912 genes were more abundantly represented and 7,700 were less abundant in ER than in LL.

There were apparent differences in the proportions of expressed genes unique to ER and LL. A total of 52,298 genes were represented in the combined endometrial DGE profiles. The proportions of genes expressed uniquely in ER and LL were 13.5% (7,060/52,298) and 40.3% (21,066/52,298), respectively; the remaining genes were shared by the transcriptomes. Of the total number of genes expressed, 1.53% (800/52,298) and 1.84% (970/52,298) had an expression level of >0.01% in the ER and LL transcriptomes, respectively.

### Gene Ontology (GO) and signalling pathway analysis

**Figure 2:**
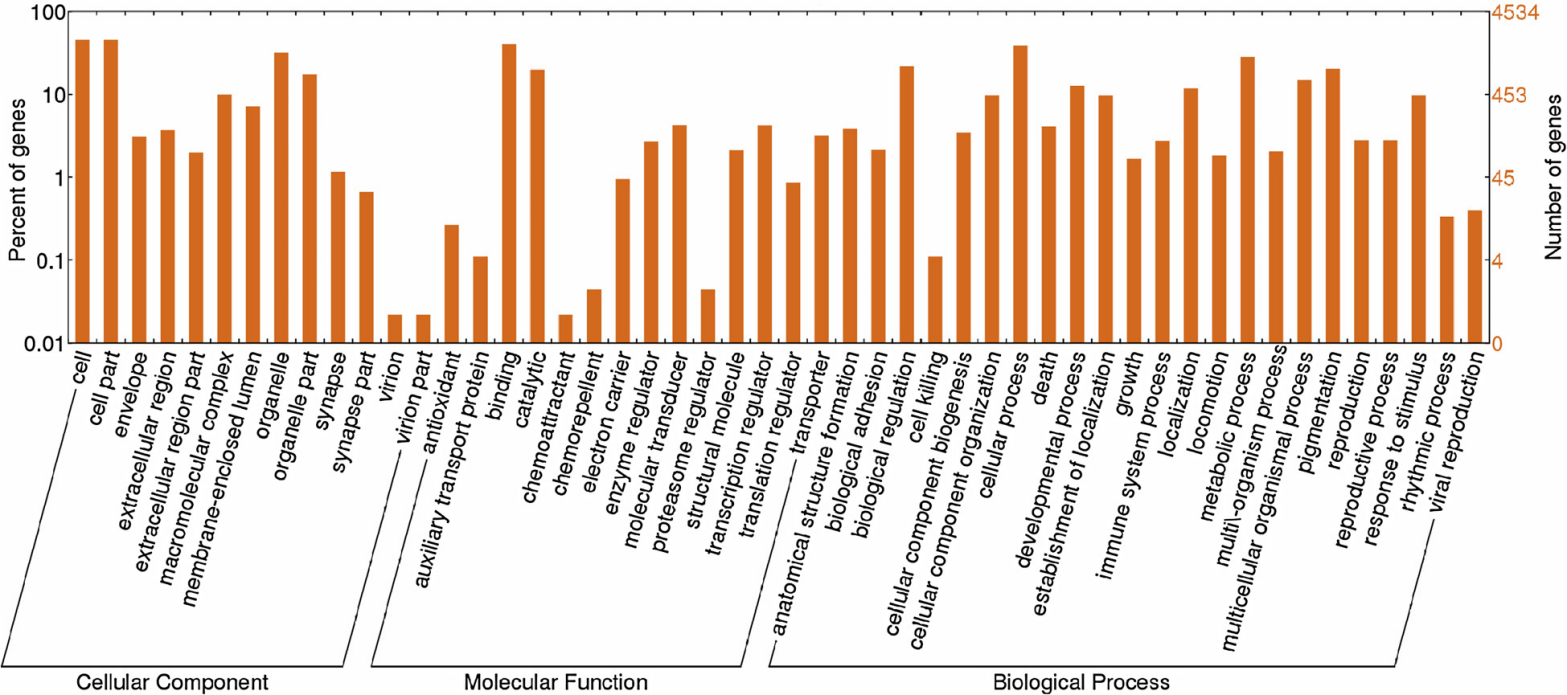
**GO analysis of differentially expressed genes between the two breeds.** The differentially expressed genes are classified into three categories: cellular component, molecular function, and biological process. The left axis shows the percentage of genes in a category, and the right axis the number of genes.

GO is an international standard system of classification for the comprehensive description of the properties of genes and their products. It was used to classify all the genes expressed in ER and LL endometrium into one of three groups according to their biological process, cellular component, and molecular function (Additional file 8: Figure S3 and Figure 2). GO analysis of all the genes expressed in this period revealed that the overall genomic expression profiles were very similar between ER and LL, but differences were detected in specific aspects such as cellular synapses and proteasome regulation. The GO annotation (Figure 2) indicated that the differentially expressed genes were involved in many processes, such as reproduction, growth, cellular component biogenesis and organization, biological adhesion, and immune function.

To identify the metabolic and signal transduction pathways in which the differentially expressed genes are likely to be involved, we performed pathway analysis on the basis of the Kyoto Encyclopedia of Genes and Genomes (KEGG) pathway database using an ultra-geometric test. In total, 4,006 differentially expressed genes had KEGG pathway annotations. As shown in Additional file 9: Table S6, the significant signaling pathways included steroid biosynthesis, oxidative phosphorylation, basal transcription factors, and the transcription machinery.

### qPCR analysis

**Figure 3:**
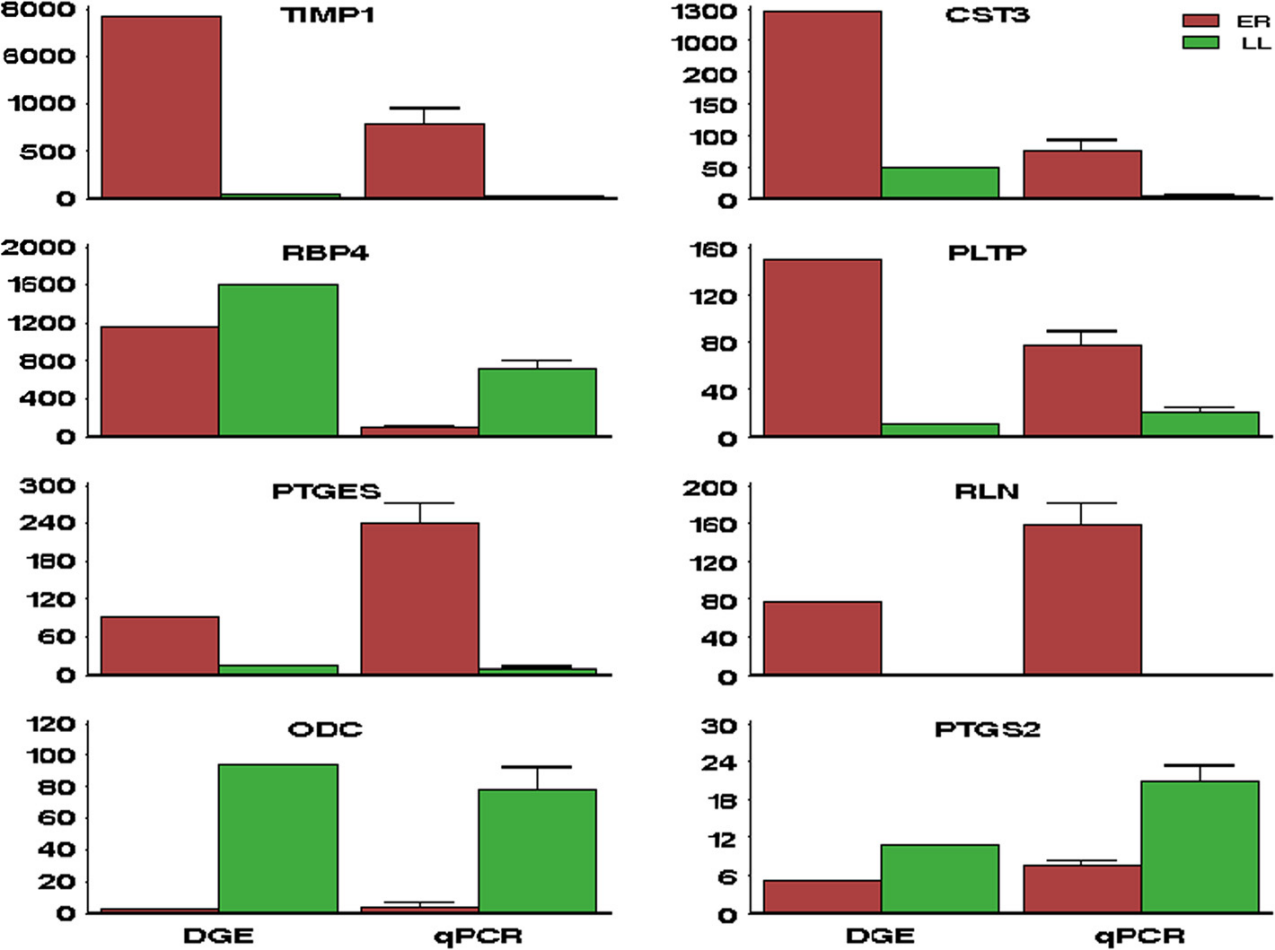
**Validation of the DGE results by qPCR.** This figure illustrates that the qPCR results are consistent with those of DGE. The qPCR results were normalized to the expression level of *RPS20*. See Additional file 7: Table S5 for DGE results, and Additional file 10: Table S7 for details of these genes. DGE results: *FDR* < 0.01 for all listed genes between breeds. qPCR results: error bars represent SE; the expression levels of all listed genes differ highly significantly (*P* < 0.01) between breeds.

Quantitative real-time RT-PCR (qPCR) was performed on eight genes to confirm the patterns of differential gene expression between ER and LL pigs. The detailed information about these genes was listed in Additional file 10: Table S7. This set included five genes that showed increased DGE representation in ER (*TIMP1*, *CST3*, *PLTP*, *PTGES*, and *RLN*) and three genes with lower DGE representation in ER (*RBP4*, *ODC*, and *PTGS*2). As shown in Figure 3, qPCR validated the results of the DGE analysis in all cases.

### Expression analysis of candidate genes for embryonic survival

Quantitative trait loci (QTL) related to the embryonic survival rate in pigs have rarely been reported so far [37], probably because of the high costs of such experiments. Hence, candidate genes have been selected on the basis of their physiological functions and the results of candidate gene studies. We concentrated on maternal genes related to embryonic growth and PG synthesis. The expression levels and functions of the candidate genes are listed in Table 2.

**Table 2:** Differentially expressed genes in the endometrium that may affect sow prolificacy

**Gene**	**Expression Level (TPM)**		***FDR***	**Function**
	**ER**	**LL**		
*PTGS1*	14.5	3.64	1.37E-06	− Converts arachidonic acid to PGH_2_[28,38].
				− Rate-limiting enzymes in PG synthesis [38].
				− Essential to reproduction [39-43].
*PTGS2*	5.22	10.92	1.20E-02	− The same as those of PTGS1.
*PTGES*	91.93	15.10	1.68E-12	− Converts PGH_2_ to PGE_2_[30].
*PTGES2*	25.52	9.64	8.36E-08	− Converts PGH_2_ to PGE_2_ [44,45].
*CBR1*	1.16	0.01	1.70E-02	− Converts PGE_2_ into PGF_2α_[46].
*CBR2*	5.22	141.9	1.17E-136	− Converts PGE_2_ into PGF_2α_ according to KEGG pathway.
*RBP4*	1160.03	1609.79	7.06E-67	− Transports vitamin A to the embryos [47], thereby affecting the growth and development of the embryos [48].
*UF*	102.37	1175.01	0.00E-00	− Transports iron to the embryos [49].
*IGF1*	0.58	14.01	4.99E-13	− Additively has metabolic, mitogenic and differentiation actions and are essential for prenatal growth of the conceptus [50,51].
				− Control and differentiation of the uterus for blastocyst implantation [52].
*IGF2*	13.92	2.18	2.83E-05	− The same as those of IGF1.
*HB-EGF*	0.01	1.64	3.62E-02	− Blastocyst growth, and trophoblast outgrowth [53] and development [54].
*FGF7*/*KGF*	8.70	71.31	3.07E-48	− Proliferation and differentiation of trophectoderm [55].

See Additional file 7: Table S5 for details of all the differentially expressed genes. Numbers in square brackets are the numbers of the papers in the reference list.

## Discussion

In this study, we generated the endometrial expression profiles and identified the genes differentially expressed in GD12 ER and LL endometrium. The results in this paper will be valuable for future studies on the identification of major genes for embryonic survival.

### The genes for growth factors and nutrient-delivery proteins

The secretion of uteroferrin (UF) is not responsive to the plasma levels of iron [56], thus it is speculated that the iron supply to the embryos during the peri-implantation period is determined by genotypes. *Retinol-binding protein 4* (*RBP4*) is significantly associated with litter size in German Landrace pigs (*P* < 0.05) [57]. Receptors for HB-EGF [58], KGF [59], IGF1 [60,61], and IGF2 [62] are all expressed by porcine embryos on GD12. Studies have shown that IGF1 promotes embryonic growth in response to the nutrient supply [63,64], while IGF2 may regulate the supply of maternal nutrient to conceptus [65]. The expression levels of the five genes (*RBP4*, *UF*, *HB-EGF*, *KGF*, and *IGF1*) and *IGF2* in LL versus ER pigs were significantly up-regulated and down-regulated (Table 2), respectively. Vallet et al. (1998) [36] reported that expressions of UF and RBP were lower in pregnant Meishan endometrium than in White crossbred. The GO molecular function classification showed that the differential genes were associated with growth (Figure 2). The expression patterns and the physiological functions of these genes (Table 2) indicated that the endometrium of ER pigs had a lower growth-promoting ability to embryos than that of LL pigs. The above results can partially explain the phenomenon that embryos in uteri of Taihu sows grow slower than those in the uteri of Western sows.

*IGF1* was expressed significantly higher in LL endometrium than in ER endometrium (Table 2). IGF1, rather than IGF2, is known to induce estrogen synthesis by stimulating expression of aromatase in the conceptus [60,66]. Aromatase is the rate-limiting enzyme in estrogen synthesis in the pig conceptus [67]. Therefore, it is very likely that ER embryos secret less estrogen than LL embryos, which will contribute to the higher embryonic survival rate in ER pigs. Moreover, IGF2 increases the permeability of the placenta in mice [65,68], and thus a higher level of *IGF2* in ER endometrium (Table 2) may improve the placental efficiency.

### The genes in the prostaglandin (PG) synthetic pathway

PG synthesis in endometrum, especially the PGE_2_:PGF_2α_ ratio, is crucial for the establishment and maintenance of pregnancy in pigs [27,31,46]. The high PGE_2_:PGF_2α_ ratio may be a beneficial factor for large litter size in Meishan sows [31,33]. The expressions of the genes, *PTGS1*/*PTGS2*, *PGES*/*PGES2* and *CBR1*/*CBR2*, play critical roles in the PG synthesis.

PTGS1 and PTGS2 are rate-limiting enzymes in PG synthesis pathway [38]. The expression level of *PTGS2* is higher than that of *PTGS1* in the endometrium of LL pigs on GD12 (Table 2), which is consistent with other studies using Western pigs [38,69]; while the expression level of *PTGS2* is lower than that of *PTGS1* in ER sows (Table 2). In Western sows, PTGS2 is the primary enzyme involved in elevated PG synthesis [38,69], whereas PTGS1 may perform this function in ER sows according to our results. It has been demonstrated that both the mRNA and the protein of PTGS2 have shorter half-lives than those of PTGS1 [70]. Hence, the higher *PTGS1* expression can contribute to the larger capacity for PG synthesis in ER pigs on GD12.

The convert of PGH_2_ to PGE_2_ in PG synthesis is catalyzed by PTGES [30] and PTGS2 [44,45]. The higher expression of *PTGES* and *PTGES2* in ER endometrium (Table 2) is helpful for the higher PGE_2_, which will be contribute to the higher ratio of PGE_2_ to PGF_2α_ on GD 12.

In Western breeds, expression of *CBR1* has been examined [31] but *CBR2* neglected. Although the levels of endometrial CBR1 on GD12 and GD14 did not differ [31], the ratio of PGE_2_ to PGF_2α_ on GD14 was higher than that on GD12 [33]. CBR2 may play a role in the conversion of PGE_2_ into PGF_2α_ according to our results and KEGG pathway (http://www.genome.jp/kegg-bin/show_pathway?org_name =ssc&mapno = 00590&mapscale = 1.0&show_description = show), and that higher expression of CBR2 may decrease the ratio of PGE_2_ to PGF_2α_. In the present study, the patterns of expression of *CBR1* and *CBR2* observed in the two breeds (Table 2) suggest that the ratio in the endometrium of ER pigs be greater than that in the endometrium of LL pigs on GD12.

## Conclusions

In summary, we have described genes that are expressed differentially in the endometrium of ER and LL pigs on GD12. Compared with those in the LL pigs, the gene expression profiles in the endometrium of the prolific ER pigs are found to benefit for the establishment and maintenance of pregnancy, delay embryonic development and growth, and enhance uterine capacity via reduced estrogen secretion. The gene-driven events that are characteristic of ER pigs could contribute to the lower embryonic mortality and higher prolificacy of this indigenous Chinese Taihu breed. The data provided by this study will be useful for porcine transcriptomic studies.

## Methods

### Animal and tissue collection

All animal procedures were performed according to protocols approved by the Biological Studies Animal Care and Use Committee of Guangdong Province, China. Three LL sows (parity 3) and three ER sows (parity 3) were artificially inseminated (AI), and slaughtered on GD12. Endometrial samples were collected and stored at −80°C until RNA extraction was performed [71].

### RNA extraction and cDNA libraries construction

Total RNA was isolated from the frozen endometrium of the two breeds using the TRIzol reagent (Invitrogen). The qualified total RNA was diluted to the same concentration, and then was reverse transcribed individually to generate cDNA libraries by first-strand cDNA synthesis kit (Takara).

### Construction of reference tag library

In order to generate a reference tag library, we downloaded the *Sus scrofa* Unigene from the National Center for Biotechnology Information (NCBI, http://www.ncbi.nlm.nih.gov) (UniGene Build #36), reference cDNA library (Sscrofa9.58.cdna.all) from ENSEMBL (http://www.ensembl.org), and Tentative Consensus sequences (TCs, Release 13.0) from The Institute of Genome Research porcine index (TIGR, http://compbio.dfci.harvard.edu/tgi/). These databases were used according to a preset priority. The priority order was Unigene from NCBI, confirmatory gene/cDNA from ENSEMBL, TCs from TIGR, and novel and pseudogene predictions from ENSEMBL. The sense and antisense tags sequences of the references genes were included in the reference tag library.

### DGE library construction and tag sequencing

Equal quantities of mRNA from three LL animals were pooled as a control sample, and mRNA from three ER as treatment sample. For sequence tag preparation, the two mRNA samples (6 μg respectively) were treated with Illumina^′^s Digital Gene Expression Tag Profiling Kit [72,73]. The DGE tag libraries were anchored on the flowcell. During *in situ* amplification the single tag became clusters, which served as a template for sequencing on the Illumina Cluster Station and Genome Analyzer. Raw image data were transformed into the DGE tag sequence by base calling.

### Analysis of DGE tag sequences

Raw data were filtered by Solexa mRNA tag pipeline (the copyrights are reserved by Beijing Genomics Institute, the number of copyright registration is 2009SR05447 in China) to remove adaptors, low quality tags and tags of copy number = 1, and a clean tag library was generated. The total tags were classified according to the copy numbers in the library and their percentages in the total tags and unique tags were shown. In addition, saturation analyses of the two DGE clean tag libraries were executed to determine their overall quality.

### Mapping DGE tags

All clean DGE tags were mapped by aligning the sequences of DGE tags to the reference tag library. Unambiguous tags were annotated and ambiguous tags discarded. The clean tags corresponding to each gene were counted to quantify expression abundance of the genes. The raw expression levels were normalized to TPM [72,73]. Statistical analysis of abundance of gene expression in endometrium was preformed, and the differently expressed genes were screened [74,75]. Genes were deemed significantly differentially expressed with *P* values <0.001, false discovery rate (*FDR*) <0.001 and absolute value of log2-fold change > 2 in TPM between libraries. Genes with antisense reference tags corresponding to DGE tags were exclusively listed and annotated. The DGE tags that were unable to be mapped to the reference tag library and mitochondria were aligned to the nuclear genome to detect potential novel transcripts.

### GO analysis

The hypergeometric test was preformed to identify significantly enriched GO terms by comparing to the whole genomic background [76]. GO terms with a Q-value (i.e. Bonferroni adjusted *P* value) was less than 0.05 were defined as the significantly enriched GO terms. Furthermore, WEGO was employed to plot GO annotations of all expressed and differentially expressed genes [77].

### Pathway analysis

According to KEGG database, hypergeometric test and multiple hypotheses correction were used to classify the pathway category [76]. Pathways with a Q-value was less than 0.05 were defined as a significantly pathway enriched with differential gene expressions.

### Validation of DGE results by real-time qPCR

qPCR was employed, and eight genes were selected to verify the DGE results. The details of these eight genes are summarized in Additional file 10: Table S7. Independent cDNA from the three sows for tag sequencing was used as template in LL and ER, respectively. qPCR was preformed with SYBR® *Premix Ex Taq*™ (Takara) on Lightcycler480 (Roche). For each biological replicate, the reactions of all eight genes and one pre-selected housekeeping gene were run on one plate in triplicate for each gene to represent technical replicates. The relative expression levels were calculated with the 2^-ΔΔCt^ method [78]. We had found that *ribosomal protein S20* (*RPS20*) was the most suitable reference gene for comparison due to the stable expression between the two pig breeds [79], hence the results were normalized to the expression level of *RPS20*. The *t*-test was used to compare the levels of expression between the two breeds [80].

## Abbreviations

CBR: Carbonyl reductase; CST3: Cystatin C; DGE: Digital gene expression profile; ER: Erhualian; FDR: False discovery rate; FGF7/KGF: Keratinocyte growth factor/fibroblast growth factor-7. GD, gestation day; GO: Gene ontology; HB-EGF: Heparin-binding EGF-like growth factor; IGF: Insulin-like growth factor; KEGG: The Kyoto Encyclopedia of Genes and Genomes; LL: Landrace × Large White; ODC: Ornithine decarboxylase; TIMP1: TIMP metallopeptidase inhibitor 1; PTGES: Prostaglandin E synthase; PTGS: Prostaglandin G/H synthesis; qPCR: Quantitative real-time RT-PCR; RBP4: Retinol binding protein 4; RLN: Relaxin; RPS20: Ribosomal protein S20; TPM: Tags per million; UF: Uteroferrin.

## Competing interests

The authors have declared that no competing interests exist.

## Authors’ contributions

HZ and JL conceived and designed the experiments. HZ, SW, ML, AZ, ZW, and ZZ performed the experiment and analyzed the data. HZ, SW, AZ, and JL wrote the paper. All authors read and approved the final manuscript.

## Supplementary Material

Additional file 1: Figure S1 Distribution of total clean tags and unique clean tags. The top panel displays the distribution of total clean tags and the bottom panel displays the distribution of unique clean tags. The left row shows the details of ER and right row shows the situations of LL. Click here for file

Additional file 2: Figure S2 Tag position analysis. Tag position analysis reveals the positions of tags in the gene. Click here for file

Additional file 3: Table S1 Antisense transcripts and their corresponding genes of ER. Click here for file

Additional file 4: Table S2 Antisense transcripts and their corresponding genes of LL. Click here for file

Additional file 5: Table S3 Tags mapped to nuclear genome for ER. Click here for file

Additional file 6: Table S4 Tags mapped to nuclear genome for LL. Click here for file

Additional file 7: Table S5 All differentially expressed genes. Click here for file

Additional file 8: Figure S3 GO analysis of all expressed genes in endometrium. GO analyses of all expression genes were performed according to Gene Ontology database. Click here for file

Additional file 9: Table S6 Significantly enriched pathway of differentially expressed genes. Click here for file

Additional file 10: Table S7 The details of the eight genes for qPCR. Click here for file
