# Supplementary figures and images for: Differential gene expression in the endometrium on gestation day 12 provides insight into sow prolificacy
Source: BMC Genomics. 2013 Jan 22;14:45. doi: 10.1186/1471-2164-14-45 (PMC3610143; doi:10.1186/1471-2164-14-45)

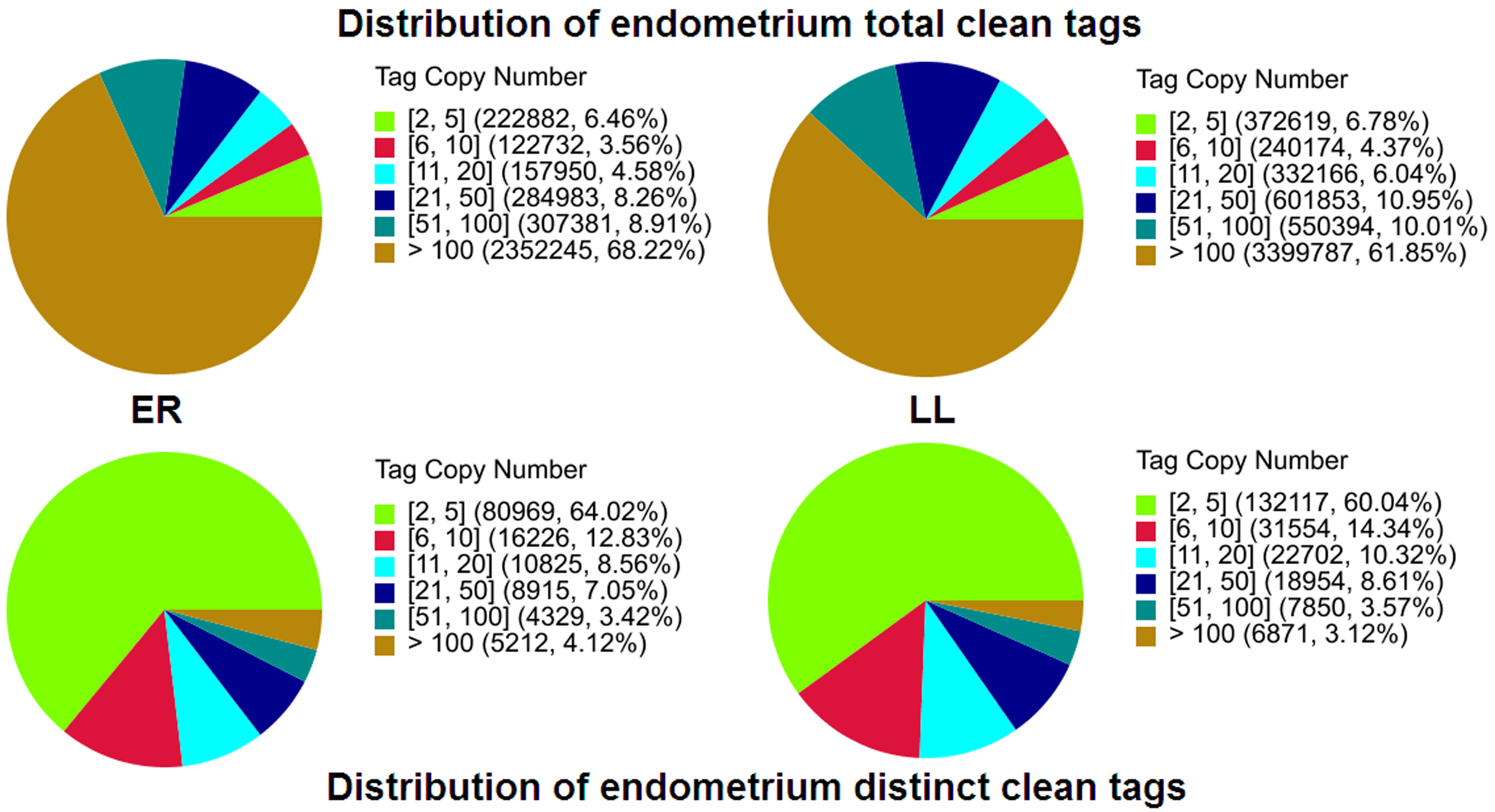

Supplement: Additional file 1: Figure S1 — Distribution of total clean tags and unique clean tags. The top panel displays the distribution of total clean tags and the bottom panel displays the distribution of unique clean tags. The left row shows the details of ER and right row shows the situations of LL. [file 1471-2164-14-45-S1.tiff]

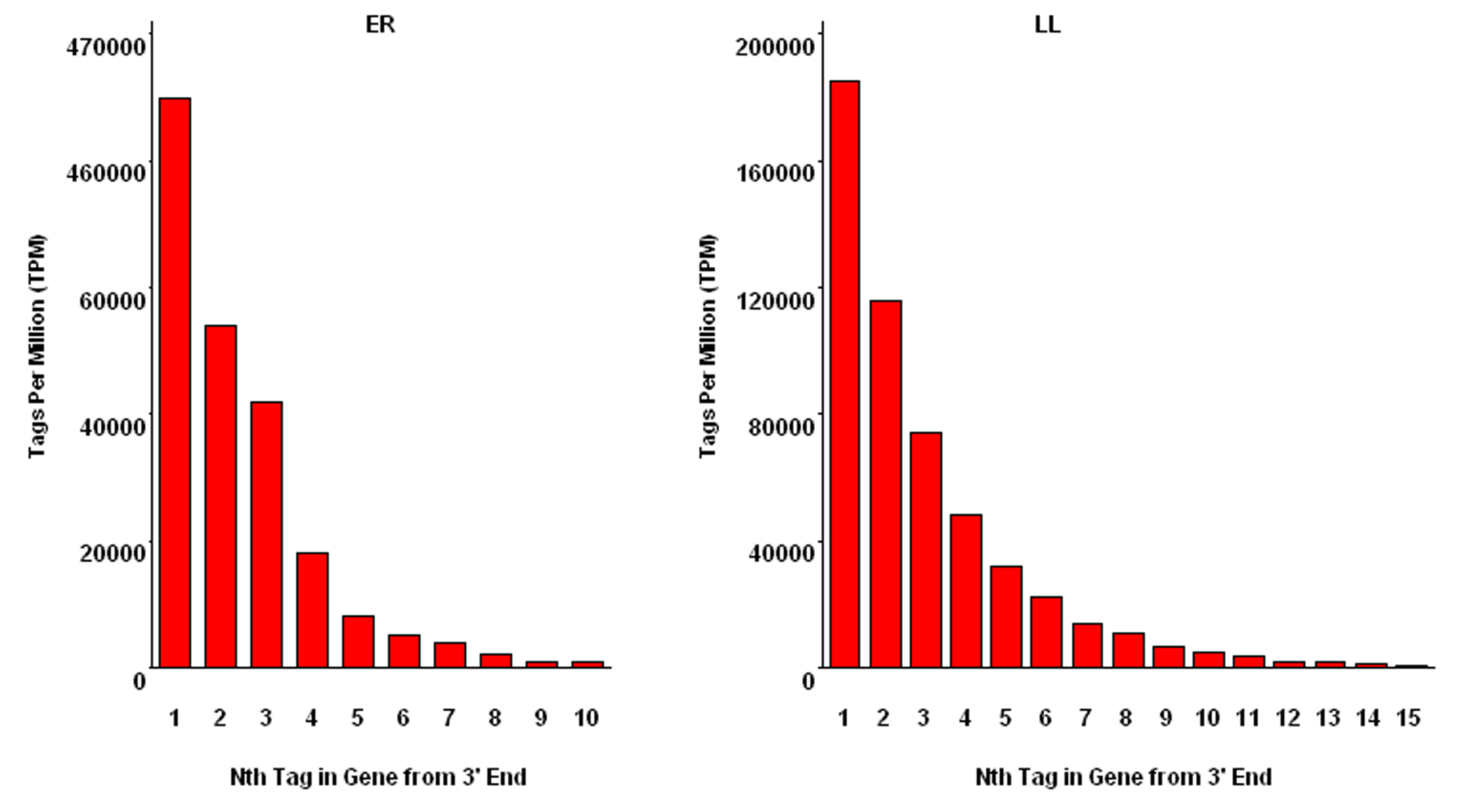

Supplement: Additional file 2: Figure S2 — Tag position analysis. Tag position analysis reveals the positions of tags in the gene. [file 1471-2164-14-45-S2.tiff]

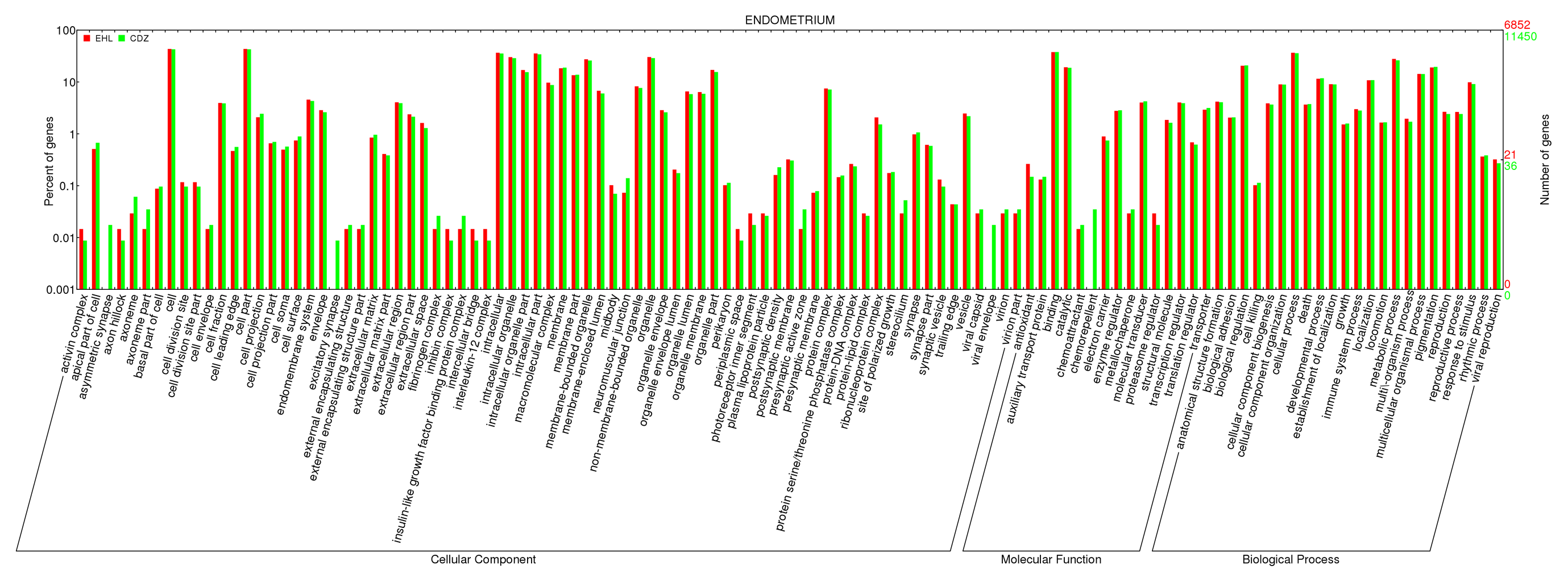

Supplement: Additional file 8: Figure S3 — GO analysis of all expressed genes in endometrium. GO analyses of all expression genes were performed according to Gene Ontology database. [file 1471-2164-14-45-S8.tiff]
